# Rol‐Transformed *Kalanchoë* Shows Altered Root Allocation and Photosynthetic Responses to Nitrogen Deficiency

**DOI:** 10.1111/ppl.71140

**Published:** 2026-10-05

**Authors:** Alessandro Esposito, Giorgia Saccardo, Yannick Valentin El Khoury, Natalia Prieto Vidal, Ondřej Vrobel, Petr Tarkowski, Martina Paponov, Jwan Goreal, Kai Lønne Nielsen, Ivan A. Paponov

**Affiliations:** ^1^ Department of Food Science Aarhus University Aarhus Denmark; ^2^ Department of Agricultural, Food and Forest Sciences (SAAF) University of Palermo Palermo Italy; ^3^ Czech Advanced Technology and Research Institute (CATRIN) Palacký University Olomouc Olomouc Czech Republic; ^4^ Czech Agrifood Research Center, Genetic Resources of Vegetables and Special Crops Olomouc Czech Republic; ^5^ Department of Biochemistry, Faculty of Science Palacký University Olomouc Olomouc Czech Republic; ^6^ Queen Genetics/Knud Jepsen A/S Hinnerup Denmark; ^7^ Department of Plant and Environmental Sciences University of Copenhagen Taastrup Denmark

**Keywords:** biomass allocation, crassulacean acid metabolism, *Kalanchoë*, nitrogen deficiency, phytohormones, Ri/rol transformation, root allocation, source–sink relations

## Abstract

Ri/rol transformation by *Rhizobium rhizogenes* is well known to alter plant architecture, including shoot compactness, branching and root development. However, its significance for plant responses to nitrogen limitation remains unclear. Here, we investigated developmental, physiological and hormonal responses of an interspecific *Kalanchoë laciniata* × *K. blossfeldiana* hybrid to nitrogen deficiency using complementary pot and hydroponic experiments. Nitrogen deficiency strongly reduced leaf area and total biomass in both wild‐type (WT) and rol‐transformed (Rol) plants. Nevertheless, rol transformation consistently produced a more compact, highly branched phenotype, with increased leaf number, greater side‐shoot formation, higher specific leaf area, lower leaf dry matter content, and, in hydroponics, a striking increase in root mass allocation. Rol plants also maintained higher chlorophyll and carotenoid concentrations than WT and showed altered carbon partitioning, with lower soluble sugars but a higher relative proportion of sucrose. Under nitrogen deficiency, Rol plants maintained positive net photosynthesis and higher electron transport, whereas WT plants showed near‐complete suppression of CO_2_ assimilation despite high intercellular CO_2_ concentration, pointing to strong non‐stomatal limitation. Rol plants were characterized by lower ABA and PA concentrations, reduced auxin pools and enhanced leaf cytokinin status. Under low N, greater root allocation in Rol plants was not accompanied by reduced overall growth when root expansion was unrestricted, suggesting compensation through higher specific leaf area and maintenance of photosynthetic function. The strong shift towards root investment highlights a potential role of rol transformation in applications where belowground biomass and root development are especially relevant.

## 1 Introduction

Nitrogen (N) plays a central role in plant growth and metabolism as a core constituent of amino acids, nucleic acids, chlorophyll and numerous proteins, including key enzymes of photosynthesis (Squeri et al. 2021; Mishra et al. 2024; Comadira et al. 2015; Paponov and Engels 2003). Nitrogen availability therefore strongly influences growth, photosynthetic capacity, chlorophyll formation, carbon metabolism and biomass allocation. It affects photosynthetic enzyme content, modulates Calvin cycle activity and carbon partitioning between protein synthesis and storage compounds, and governs resource distribution between roots and shoots (Peng et al. 2024; Paponov et al. 2020; Paponov et al. 2000). Importantly, N deficiency does not merely reduce growth proportionally, but induces coordinated changes in shoot–root allocation, branching architecture, leaf structural traits, photosynthetic performance, metabolite balance and hormone signalling (Paponov et al. 2021; Sakioka and Yoneyama 2025; Li 2024; Sakakibara 2021; Xing et al. 2023). These integrated responses make N deficiency a particularly informative condition for examining how genetic or developmental modifications reshape whole‐plant acclimation at morphological, physiological and hormonal levels.

CAM plants differ from C3 and C4 species in the temporal organization of carbon fixation, with nocturnal CO_2_ uptake and organic acid accumulation supporting daytime photosynthesis under reduced stomatal opening (Winter and Smith 1996; Winter and Holtum 2014). The extent of CAM expression varies among species and environmental conditions (Winter et al. 2008; Winter 2019; Heyduk 2022). Nitrogen availability can influence CAM‐related metabolism and gas exchange. In *Kalanchoë blossfeldiana*, N deficiency increased nocturnal CO_2_ uptake, PEPC activity and organic acid accumulation, whereas in *K. lateritia*, CAM expression was highest at an intermediate reduced‐N level. CAM activity in *Kalanchoë* can also vary with N concentration and N source (Santos and Salema 1991; Ota 1988; Ota and Yamamoto 1991; Pereira et al. 2017). Thus, N availability is an important regulator of CAM‐related function in *Kalanchoë*, but its interaction with other developmental regulators at the whole‐plant level remains poorly understood.

Ri/rol transformation is based on infection with *Rhizobium rhizogenes* (syn. *Agrobacterium rhizogenes* ) and transfer of root‐inducing plasmid T‐DNA into the plant genome. The transferred T‐DNA contains *rol* genes, and in some Ri plasmid types also aux‐related genes, which can induce hairy‐root formation and, after regeneration, give rise to whole plants with the characteristic Ri phenotype. Across many species, Ri/rol transformation has been associated with compact growth, reduced internode elongation, altered branching, modified leaf morphology and enhanced adventitious or lateral root formation (Denaeghel et al. 2020; Desmet, Dhooghe, de Keyser, Quataert, et al. 2020; Desmet et al. 2024). These developmental effects are generally interpreted as consequences of altered hormonal sensitivity and/or hormonal balance, particularly involving auxin‐ and cytokinin‐related regulation (Mauro and Bettini 2021). The effects of *Ri/rol* transformation are also relevant for plant nutrient acquisition and metabolism. Root architecture, root‐to‐shoot allocation and hormonal signalling are major determinants of mineral nutrient uptake and nutrient‐use responses (Paponov et al. 1999; Giehl et al. 2014; Jia and von Wirén 2020). Therefore, the enhanced rooting capacity and altered root–shoot balance commonly observed in *Ri/rol*‐transformed plants may influence nutrient acquisition indirectly by changing the size, activity or allocation priority of the root system. In addition, *rol* genes, especially *rolB* and *rolC*, have been widely reported to modify plant metabolism, including secondary‐metabolite accumulation and stress‐related metabolic pathways (Bulgakov 2008; Mauro and Bettini 2021). These metabolic effects suggest that Ri/rol transformation can influence not only plant form, but also the internal physiological state that determines how plants acquire, assimilate and allocate resources. Nevertheless, most studies on *Ri/rol*‐transformed plants have focused on morphology, regeneration capacity, secondary metabolism or horticultural performance, whereas direct evidence of effects on nutrient acquisition, nitrogen metabolism and whole‐plant responses to nutrient limitation remains limited.

Although N deficiency is known to alter growth, photosynthesis, carbon metabolism and hormonal regulation (Paponov et al. 2021; Sakakibara 2021) and Ri/rol transformation is known to modify root development, plant architecture, hormonal regulation and metabolism (Desmet, Dhooghe, de Keyser, VAN Huylenbroeck, et al. 2020; Mauro and Bettini 2021), their interaction remains poorly understood. In particular, it is unclear whether rol transformation modifies biomass allocation under low N, the maintenance of photosynthetic function, carbon partitioning and malate dynamics, or hormonal responses to N limitation. This question is especially relevant in CAM plants, where developmental regulation, gas‐exchange patterning and carbon metabolism are closely interconnected (Winter 2019; Heyduk 2022). Resolving this gap is necessary to determine whether rol transformation affects only plant architecture or also the broader physiological framework through which CAM plants acclimate to N deficiency.

To address this question, we compared wild‐type (WT) and rol‐transformed plants of interspecific *Kalanchoë laciniata* × *K. blossfeldiana* hybrid under control and reduced N supply in two complementary systems, pot culture and hydroponics. We assessed responses at multiple levels, including growth, biomass allocation, leaf structural traits, pigment composition, gas exchange, carbon‐related metabolites and hormone profiles. We hypothesized that rol transformation modifies the response to N deficiency by reshaping plant architecture, biomass allocation, photosynthetic performance, carbon partitioning and hormonal balance, thereby producing a developmental and physiological response pattern distinct from that of WT plants.

## 2 Methods

### 2.1 Plant Materials and General Experimental Conditions

The experiments were conducted during 2023–2024 at the Department of Food Science, Aarhus University, Agro Food Park, Denmark, under controlled‐environment conditions. Two *Kalanchoë* interspecific hybrid genotypes were used in this study: the non‐transformed line 2006‐0199 AAE, hereafter referred to as the wild type (WT), and the Ri‐transformed line 4006‐0199 S1, hereafter referred to as Rol. The Rol line originated from breeding material developed through transformation with a wild‐type strain of *Agrobacterium rhizogenes* A4 (ATCC43057) and subsequent selection of Ri lines with a low copy number, as described previously in the related patent and background study (Nielsen 2021; Christensen et al. 2008). The transformed status of line 4006‐0199 S1 was additionally confirmed by qPCR amplification of the TL‐DNA genes *rolB* and *rolC*, using line 2006‐0199 as a negative control and *A. rhizogenes* strain A4 as a positive control.

### 2.2 Basic Nutrient Solution

In both experiments, plants in the control treatment received a custom low‐ionic‐strength nutrient solution prepared with distilled water. The formulation was adapted from the modified Hoagland‐type solution previously used in hydroponic experiments with medicinal plants (Paponov et al. 2023), but macronutrient concentrations were reduced for the present Kalanchoë experiment. This lower ionic strength was selected because Kalanchoë is a slow‐growing succulent CAM species with lower biomass accumulation during the experimental period than fast‐growing herbaceous C_3_ crops commonly cultivated in standard Hoagland‐type solutions. The solution contained 0.5 mM Ca(NO_3_)_2_, 0.1 mM KH_2_PO_4_, 0.5 mM K_2_SO_4_ and 0.6 mM MgSO_4_. Micronutrients consisted of 20 μM Fe, 1.25 μM Mn, 1.5 μM Zn, 25 μM H_3_BO_3_, 0.5 μM Cu, 0.175 μM Na_2_MoO_4_·2H_2_O and 50 μM KCl. The pH of the nutrient solution was maintained between 5.0 and 6.5.

### 2.3 Experiment 1. Pot Experiment

#### 2.3.1 Plant Cultivation and Growth Conditions

Vegetative side shoots of similar size were collected from stock plants propagated at Aarhus University from genotypes originally provided by Knud Jepsen A/S, Hinnerup, Denmark and transplanted individually into brown 0.5‐L plastic pots filled with a peat‐based substrate amended with clay (Substrate No. 2, Pindstrup Mosebrug A/S). No additional N was incorporated into the peat mix at planting. To reduce evapotranspiration and promote root initiation, pots were covered with plastic film for 7 days after planting. Plants were initially cultivated in a climate‐controlled greenhouse under day/night temperatures of 25/18°C and a relative humidity of 55%. Supplemental lighting was provided by programmable LED lamps (FL300 Grow, blue:red ratio 14:86; Senmatic A/S) under an 18 h photoperiod. Lamps were switched on when natural irradiance was insufficient to maintain a photosynthetic photon flux density (PPFD) of approximately 400 μmol m^−2^ s^−1^. Ambient CO_2_ concentration was maintained at 400 ppm.

At 4 weeks after cutting, plants were transferred to two climate‐controlled walk‐in chambers (FytoScope FS‐WI, Photon Systems Instruments, spol. s r.o.), where environmental conditions were adjusted to match those in the greenhouse as closely as possible. Within each chamber, both genotypes (WT and Rol) were grown under two nitrogen regimes: control nitrogen supply and nitrogen deficiency (−N). DroughtSpotter systems (Phenospex) were used to manage irrigation during the treatment period. The target weight, including pot, substrate, plant and water, was initially set at 380 g and adjusted weekly in response to increasing plant biomass and evapotranspiration demand.

#### 2.3.2 Nitrogen Treatments in the Pot Experiment

Control plants received the basic nutrient solution described above. In the nitrogen‐deficient treatment, the same nutrient solution composition was used except that Ca(NO_3_)_2_ was replaced by CaSO_4_. This substitution was chosen to maintain Ca supply while removing nitrate‐N from the solution. Thus, no nitrogen was added during the treatment period, and plants relied on residual nitrogen present in the peat‐based substrate.

Because CaSO_4_ was used as the Ca source in the nitrogen‐deficient treatment, sulphate‐S supply was higher in the nitrogen‐deficient solution than in the control solution. Therefore, sulphur was not fully balanced between the two nitrogen treatments. This approach was selected to avoid confounding N deficiency with Ca deficiency, but the possible contribution of altered sulphate availability to plant responses cannot be completely excluded.

#### 2.3.3 Gas Exchange, Time‐Resolved Leaf Sampling and Final Harvest

Photosynthetic measurements were performed only in the pot experiment. Gas exchange and chlorophyll fluorescence parameters were measured 17 days after treatment initiation, between 10:00 and 16:00 h, using a GFS‐3000 system equipped with an LED Array/PAM Fluorometer 3057‐FL (Heinz Walz GmbH). The measured parameters included net photosynthetic rate (P_𝑁_), stomatal conductance (g𝑠), intercellular CO_2_ concentration (C𝑖) and electron transport rate (ETR). The flow rate was set to 750 mL min^−1^, reference CO_2_ concentration to 400 ppm and irradiance in the leaf chamber to 400 μmol m^−2^ s^−1^.

Leaf samples for time‐resolved biochemical analyses were collected on day 20 after treatment initiation from the same plants at two sampling times: the end of the photoperiod (EOP; 24:00 h) and the beginning of the following photoperiod (BOP; 06:00 h). Sampling was initiated at EOP to minimize the potential effect of tissue wounding during the light period. For each plant, one fully developed leaf was selected, and a small tissue sample (ca. 1 g fresh weight) was collected from one side of the leaf at EOP. At BOP, a second tissue sample was collected from the opposite side of the same leaf. These paired samples were immediately frozen in liquid nitrogen and stored at −80°C until analysis of pigments and carbon‐related metabolites.

After 28 days of treatment, plants were harvested by cutting at the base, and growth and morphological parameters were determined. Leaves were separated from stems and weighed to determine fresh weight. Total leaf area was measured using a LI‐3100 Area Meter (LI‐COR). A representative leaf subsample of approximately 1.0–1.5 g fresh weight was collected at harvest for determination of dry matter, total nitrogen and nitrate concentration. The remaining leaves and stems were dried in an oven at 65°C for 6 days to constant weight for dry weight determination.

### 2.4 Experiment 2. Aeroponic Rooting and Hydroponic Cultivation

#### 2.4.1 Propagation and Rooting Conditions

Axillary shoots of WT and Rol were excised from mother plants grown under long‐day conditions in a greenhouse at the Department of Food Science, Aarhus University. For propagation, shoot cuttings were transferred to an aeroponic rooting system consisting of plastic trays and boxes placed in a growth chamber. During rooting, environmental conditions were maintained at 23/18°C day/night temperature, 85%–90% relative humidity, and a PPFD of 100 μmol m^−2^ s^−1^. During the initial establishment phase, cuttings were covered to reduce evapotranspiration and promote rooting. After 10 days, the covers were removed, and plants were grown under a light intensity of 200 μmol m^−2^ s^−1^.

#### 2.4.2 Initial Harvest for Growth Analysis

After 20 days in the aeroponic system, when roots were sufficiently developed, representative plants were harvested for the determination of stem, leaf and root fresh and dry weights as well as leaf area. For dry weight, plant material was dried at 100°C for 10 min and subsequently at 65°C to constant weight. These data were used to calculate relative growth rate (RGR) and net assimilation rate (NAR).

#### 2.4.3 Hydroponic Cultivation and Nitrogen Treatment

The remaining rooted cuttings were transferred to a hydroponic cultivation system and subjected to two nitrogen regimes: control nitrogen supply and reduced nitrogen supply. Plants were grown at 23/18°C (day/night) temperature, 70% relative humidity and a PPFD of 400 μmol m^−2^ s^−1^.

Control plants received the basic nutrient solution described above. In the reduced‐nitrogen treatment, Ca(NO_3_)_2_ was replaced by CaSO_4_ to maintain Ca supply while strongly reducing nitrate availability. The remaining macro‐ and micronutrient concentrations were the same as in the control solution. Nitrogen supply in the reduced‐N treatment was adjusted to approximately 33% of the amount taken up by plants under optimal N supply to avoid excessively severe N deficiency while still inducing a clear physiological response to N limitation. Nitrate concentration in the control nutrient solution was monitored daily using a LAQUAtwin NO_3_‐11S meter (Horiba Scientific). When the nitrate concentration fell below 0.5 mM, nitrate was replenished to maintain non‐limiting nitrogen conditions.

As in the pot experiment, replacing Ca(NO_3_)_2_ with CaSO_4_ resulted in higher sulphate‐S supply in the reduced‐N treatment than in the control treatment. This imbalance was unavoidable within the selected formulation because Ca supply was kept constant while nitrate‐N was reduced. Therefore, treatment effects are interpreted primarily as responses to reduced nitrogen availability, while acknowledging that differences in sulphate availability may have contributed to some metabolic responses.

#### 2.4.4 Final Harvest and Sampling

Plants were harvested after 21 days of hydroponic cultivation. Roots, leaves and side shoots were separated from the stem. Leaf area, leaf number, side shoot number and fresh weights of leaves, side shoots, stems and roots were recorded.

Leaf and root samples (1.0–1.5 g fresh weight, when available) were collected separately, immediately frozen in liquid nitrogen and stored at −80°C until analysis of non‐structural carbohydrates, pigments, nitrogen and hormones. Subsequently, the remaining plant material was dried in an oven at 65°C for 6 days before dry weight determination.

### 2.5 Chemical Analyses

#### 2.5.1 One‐Step Extraction of Pigments and Soluble Sugars

Metabolites were extracted from frozen samples using a two‐phase extraction method described by Salem et al. (2016). Samples were lyophilized for approximately 48 h using a BK‐FD10S freeze dryer (BIOBASE) and the dried material was ground to a fine powder using a Star‐Beater with 5 mm metal balls at 29 Hz for 3 min. A 20 mg aliquot of powder was weighed into a 2 mL Eppendorf microcentrifuge tube and extracted according to Salem et al. (2016).

Briefly, 1 mL of pre‐cooled (−20°C) methyl *tert*‐butyl ether (MTBE):methanol (3:1, v/v) was added to each sample. Samples were vortexed for 1–2 min and incubated on a shaker at 100 rpm for 45 min at 0°C–5°C, followed by sonication in an ice‐cold bath (USC300TH) for 15 min. For phase separation, 650 μL of H_2_O:methanol (3:1, v/v) was added and samples were vortexed again for 1–2 min. After centrifugation at 17,000 × g and 4°C for 15 min, the non‐polar phase and the semi‐polar phase were separated. Aliquots of both phases and the insoluble pellet were stored at −20°C. In total, approximately 750 μL of non‐polar MTBE extract and 900 μL of semi‐polar water:methanol extract were obtained.

#### 2.5.2 Quantification of Pigments

Chlorophyll *a*, chlorophyll *b*, total chlorophyll and total carotenoids were determined spectrophotometrically from the non‐polar (MTBE) phase according to Salem et al. (2016). Immediately after extraction, the upper non‐polar phase was diluted 20‐fold with 100% methanol and three technical replicates of 200 μL were pipetted into a 96‐well plate. Absorbance was measured at 470, 652 and 665 nm using a Multiskan FC Microplate Photometer (Thermo Scientific). A MTBE:methanol mixture (1:20, v/v) was used as blank. To adapt the Lichtenthaler equations to the microplate format, absorbance values were corrected for path length according to Lichtenthaler (1987). For 200 μL sample volume, a path length correction factor of 0.51 was applied, following Warren (2008). Blank values were subtracted before calculating pigment concentrations.

#### 2.5.3 Analysis of Non‐Structural Carbohydrates

Glucose, fructose and sucrose in the semi‐polar phase were quantified by HPAEC‐PAD (ICS‐6000, Dionex). Samples were diluted 1:10 in Milli‐Q water and filtered through 0.45 μm nylon filters before chromatographic analysis. Sugars were analysed with an ICS6000 HPAEC‐PAD system (Thermo Fisher Scientific) equipped with a Single Pass/Double Pass pump operating in single‐pass mode, an AS‐AP autosampler, a 10 μL injection loop and an ICS 6000 electrochemical detector. The PAD detector was operated with a gold working electrode and an AgCl reference electrode. Sugars were separated at 25°C on a Dionex CarboPac PA‐1 column (2 × 250 mm) connected to a Dionex CarboPac PA‐1 Guard column (2 × 50 mm) (Thermo Scientific) at a flow rate of 0.25 mL min^−1^. Neutral sugars were analysed in isocratic mode using 200 mM sodium hydroxide as eluent. External calibration curves for fructose, glucose and sucrose (1–100 mg L^−1^) were used for quantification. The system was controlled using Chromeleon 7.2 SR4 software (Thermo Scientific).

#### 2.5.4 Starch Analysis

Starch content was determined enzymatically by measuring glucose released after hydrolysis of the insoluble pellet remaining after liquid extraction, following Salem et al. (2016). The pellet was detached from the 2 mL Eppendorf tube and transferred to a 5 mL screw‐cap tube. It was then washed twice with 1.5 mL of 80% ethanol and centrifuged at 3000 × g for 15 min after each wash. A third wash was performed with 3 mL of 80% ethanol, after which the supernatant was discarded. Starch hydrolysis was then carried out using α‐amylase (Sigma A4582) and α‐amyloglucosidase from *Aspergillus niger* (11,202,367,001, Roche), following the general procedures of Hendrix (1993) and Zhao et al. (2010) and the manufacturers' instructions. After hydrolysis, the tube content was adjusted to 4 mL with deionized water (±0.05 g), mixed, centrifuged at 3000 × g for 10 min and the supernatant was collected. Before glucose determination, the supernatant was diluted 20‐fold with deionized water.

#### 2.5.5 Total Nitrogen, Nitrate, Malate, Total Polyphenols and Total Flavonoids

Total nitrogen content was analysed using a Gerhardt N‐Realyzer. For each sample, 50 mg of dried homogenized material from the high‐nitrogen treatment or 100 mg from the low‐nitrogen treatment was used. Nitrate concentration was determined colorimetrically in plant extracts using the salicylic acid method (Cataldo et al. 1975). Malate was quantified using a commercial enzymatic colorimetric assay kit (Malate Assay Kit, Sigma‐Aldrich, MAK067‐1KT) according to the manufacturer's instructions. Total flavonoid content was quantified by the aluminium chloride colorimetric assay and expressed as quercetin equivalents. Total polyphenol content was quantified by the Folin–Ciocalteu colorimetric assay and expressed as gallic acid equivalents (GAE).

#### 2.5.6 Hormone Analyses

Plant hormones were quantified using an isotope dilution LC–MS/MS method. Endogenous cytokinin content was analysed as previously described by Glanz‐Idan et al. (2022). Stress‐related hormones (ABA, IAA and their metabolites) were analysed according to Vrobel et al. (2024). Cytokinin standards, ABA and D6‐ABA were purchased from OlChemIm, whereas DPA, PA, NeoPA, D3‐DPA and D3‐PA were obtained from NRC. Both analyses were performed using a NEXERA X2 modular liquid chromatography system coupled to an MS‐8050 mass spectrometer (Shimadzu) via an electrospray ionization interface. Raw data were processed using LabSolutions software version 5.97 SP1 (Shimadzu). Hormone data were log‐transformed prior to statistical analysis.

### 2.6 Statistical Analysis

Statistical analyses were performed in R (R Core Team, version 4.5.0). For traits measured once per plant at harvest, data were analysed by two‐way ANOVA with nitrogen treatment and genotype as fixed factors. Because each factor had two levels, main effects were interpreted directly from the ANOVA. Tukey HSD comparisons among treatment means were performed only when the nitrogen × genotype interaction was significant.

For pigment and carbon‐related metabolite traits measured at both the end of the photoperiod (EOP) and the beginning of the following photoperiod (BOP) on the same plants, sampling time was treated as a repeated within‐plant factor. These data were analysed using linear mixed‐effects models with nitrogen treatment, genotype, sampling time and their interactions as fixed effects, and plant identity as a random effect. Significance of fixed effects was assessed from the mixed model, and the resulting effects are reported in Figure 3.

Hourly transpiration data obtained with the DroughtSpotter system were recorded repeatedly from the same plants. Therefore, diel transpiration profiles are presented descriptively as mean hourly transpiration rates ± SE over the last 5 days before harvest, without treating time or light/dark period as an independently randomized factorial treatment.

Model assumptions were checked by visual inspection of residuals for normality and homogeneity of variance.

## 3 Results

### 3.1 Rol Transformation and Nitrogen Deficiency Alter Plant Phenotype, Growth and Leaf Nitrogen Status in the Pot Experiment

Representative images showed clear genotype‐ and nitrogen‐dependent differences in plant phenotype (Figure 1). Under control N supply, Rol plants displayed stronger branching than WT, whereas N deficiency reduced shoot growth in both genotypes. The substrate surface was also more extensively covered by visible white roots in Rol plants under both N regimes, suggesting enhanced root development. These visual differences were confirmed by quantitative analysis of growth, biomass partitioning and leaf N status (Figure 2).

**FIGURE 1:**
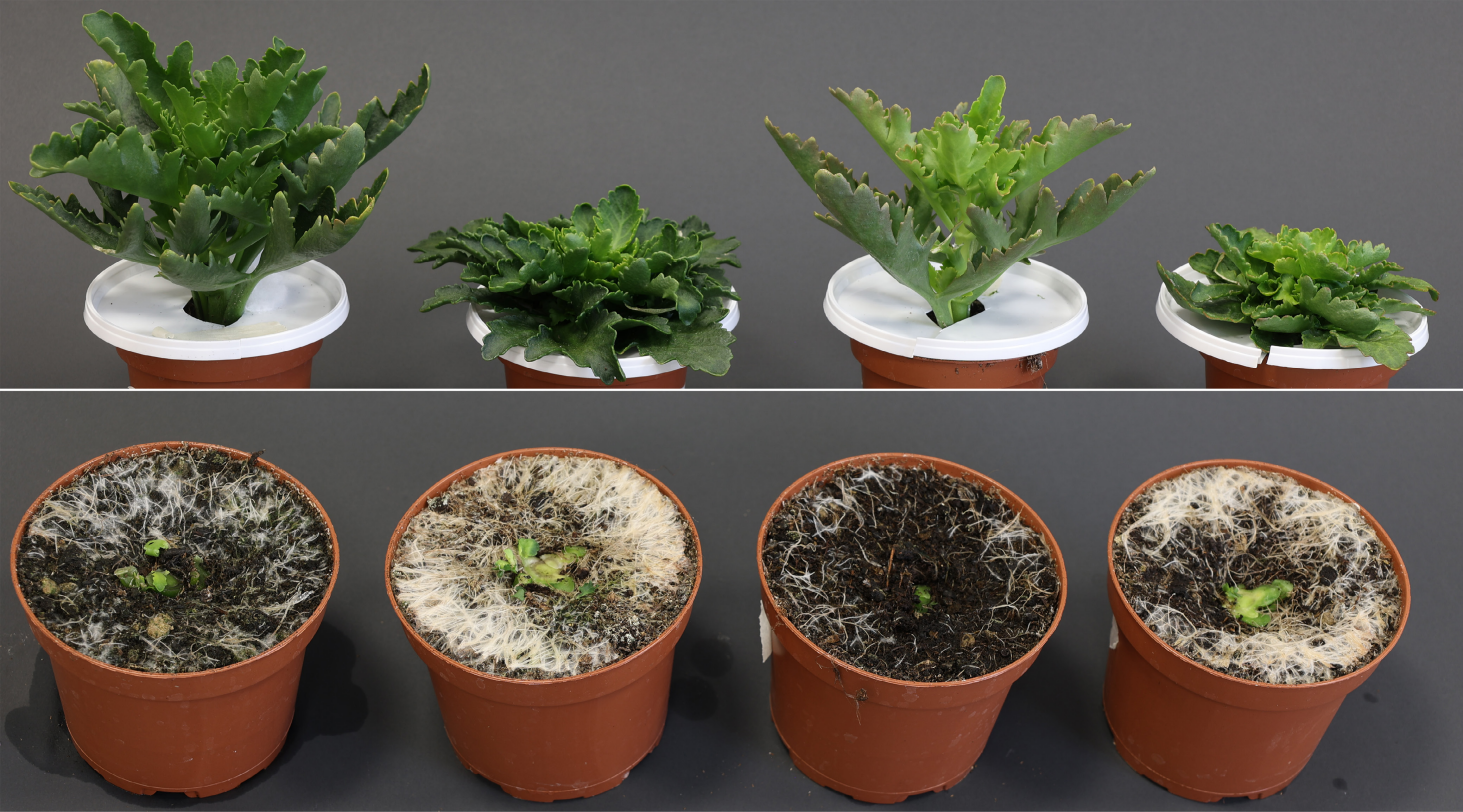
Representative shoot and root phenotypes of wild‐type (WT) and rol‐transformed (Rol) *Kalanchoë* plants grown under control nitrogen supply or nitrogen deficiency (−N). Plants are shown in the same order as in the subsequent figures: Control WT, control Rol, −N WT and −*N* Rol. The upper panel shows the shoot phenotype at harvest. The lower panel shows visible white root coverage of the substrate surface, illustrating stronger root development in Rol plants. Images are representative of *n* = 6 biological replicates per treatment.

**FIGURE 2:**
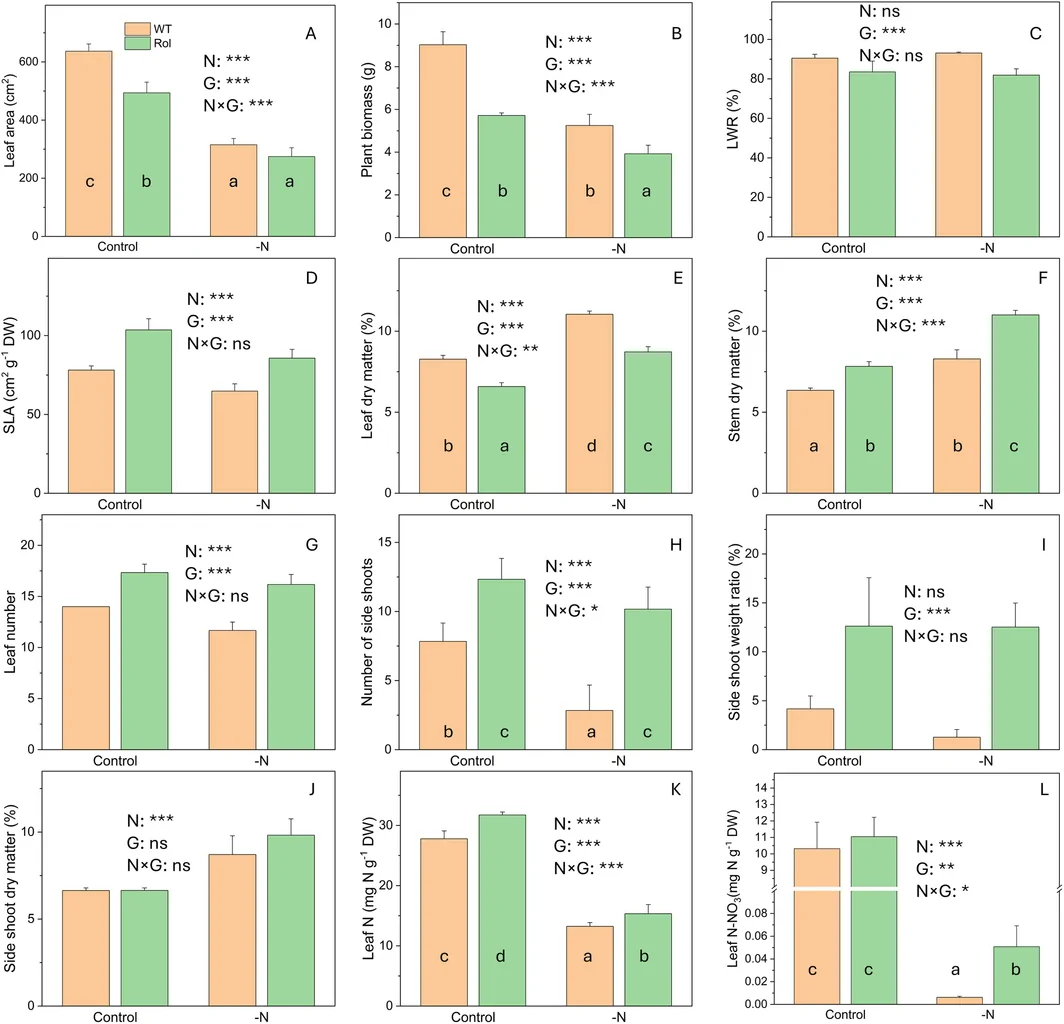
Effects of nitrogen supply and rol transformation on growth, biomass allocation, shoot architecture and leaf nitrogen status in the pot experiment. Shown are leaf area (A), total plant biomass (B), leaf weight ratio (LWR) (C), specific leaf area (SLA) (D), leaf dry matter content (E), stem dry matter content (F), leaf number (G), number of side shoots (H), side‐shoot weight ratio (I), side‐shoot dry matter content (J), leaf N concentration (K) and leaf nitrate concentration (L) in *Kalanchoë*. WT and Rol plants were grown under control and nitrogen‐deficient (−N) conditions. Bars represent means ± SE (*n* = 6 biological replicates per treatment). Different lowercase letters indicate significant differences among treatment means at *p* < 0.05 by Tukey HSD only when the *N* × G interaction was significant; otherwise, inference is based on the ANOVA main effects. Results of the two‐way ANOVA are shown in each panel: Nitrogen supply (N), genotype (G) and their interaction (*N* × G); ns, not significant; *p < 0.05, **p < 0.01, ***p < 0.001.

Nitrogen deficiency strongly reduced leaf area (by 51% in WT and 44% in Rol) and total plant biomass (by 42% and 31%, respectively). Rol plants showed lower values than WT under both N conditions, although the genotype difference was greater under non‐limiting N supply, consistent with significant *N* × G interactions (Figure 2A,B). Leaf weight ratio was lower in Rol (82%–84%) than in WT (91%–93%) and was not affected by N supply (Figure 2C). Specific leaf area decreased by approximately 17% under N deficiency in both genotypes but remained approximately 32% higher in Rol than in WT. Leaf dry matter content showed the opposite pattern, increasing by approximately 33% under N deficiency while remaining lower in Rol (Figure 2D,E). Stem dry matter content was higher in Rol and increased under N deficiency by 31% in WT and 41% in Rol (Figure 2F).

Rol transformation also markedly affected shoot architecture (Figure 2G–J). Rol plants produced approximately 31% more leaves than WT across N conditions, whereas N deficiency reduced leaf number by approximately 11% (Figure 2G). Side‐shoot number was likewise higher in Rol plants, while N deficiency caused a greater reduction in WT (64%) than in Rol (18%), consistent with a significant *N* × G interaction (Figure 2H). Side‐shoot weight ratio, defined as the proportion of total plant dry biomass allocated to side shoots, was higher in Rol (12.6% versus 2.7% in WT, averaged across N conditions) but was not significantly affected by N supply (Figure 2I). Side‐shoot dry matter content increased by approximately 40% under N deficiency, without a significant genotype effect (Figure 2J).

Leaf N concentration declined by approximately 52% under N deficiency in both genotypes but remained higher in Rol than in WT, although the absolute genotype difference was smaller under N deficiency (Figure 2K). Leaf nitrate concentration declined by more than 99% in both genotypes while remaining higher in Rol than in WT under low N (Figure 2L).

### 3.2 Pigment Composition and Carbon‐Related Metabolites Are Modified by Nitrogen Supply, Rol Transformation and Sampling Time

Because pigments and carbon‐related metabolite traits were measured at both the end of the photoperiod (EOP) and the beginning of the following photoperiod (BOP) on the same plants, sampling time was analysed as a repeated within‐plant factor (Figure 3). Total chlorophyll concentration was lower under N deficiency (by approximately 50% in WT and 40% in Rol at EOP) and at EOP than at BOP, whereas Rol plants maintained higher levels than WT (Figure 3A). The chlorophyll *a*/*b* ratio generally increased from EOP to BOP and was higher under N deficiency (Figure 3B). Carotenoid concentrations followed a similar pattern to total chlorophyll, decreasing by approximately 40% under N deficiency at EOP while remaining higher in Rol than in WT plants (Figure 3C).

**FIGURE 3:**
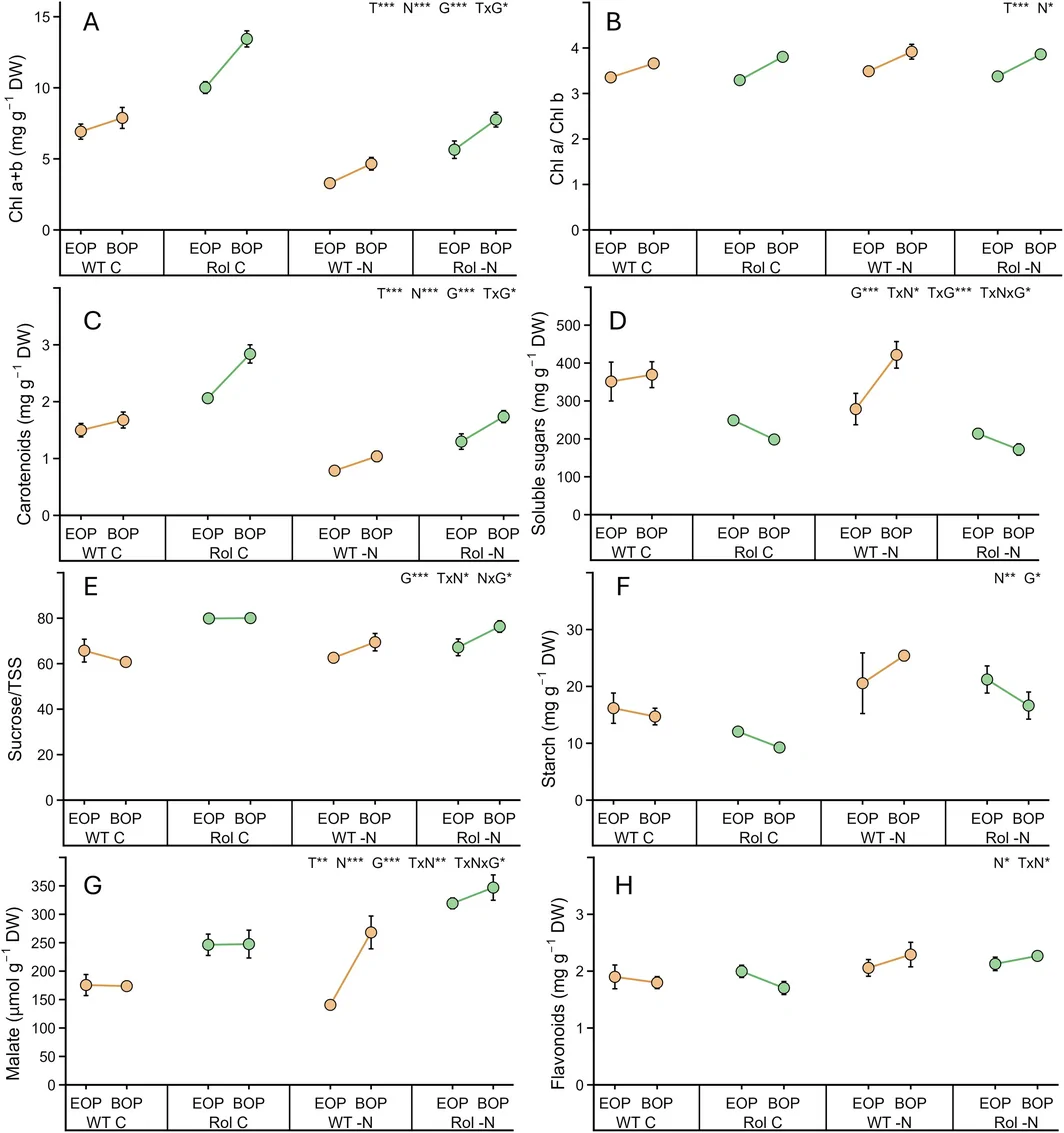
Effects of nitrogen supply, rol transformation and sampling time on leaf pigments and carbon‐related metabolites in the pot experiment. Shown are total chlorophyll (chlorophyll a + b) (A), chlorophyll a/b ratio (B), carotenoids (C), soluble sugars (D), sucrose as a proportion of total soluble sugars (E), starch (F), malate (G) and total flavonoids (H) in Kalanchoë leaves. WT and Rol plants were grown under control and nitrogen‐deficient (−N) conditions. Leaf samples were collected at the end of the photoperiod (EOP) and at the beginning of the following photoperiod (BOP). Points represent means ± SE (*n* = 6 biological replicates per treatment); lines connect EOP and BOP means within each genotype × nitrogen‐treatment combination. Sampling time was analysed as a repeated within‐plant factor, with plant included as a random effect. Significance levels from the mixed‐model analysis are shown in each panel for sampling time (T), nitrogen supply (N), genotype (G) and their interactions; ns, not significant; *p < 0.05, **p < 0.01, ***p < 0.001.

Soluble sugar concentrations were generally higher in WT than in Rol plants, but the temporal response depended on both genotype and N supply, with the strongest EOP‐to‐BOP increase (approximately 50%) occurring in WT under N deficiency (Figure 3D). The sucrose/TSS ratio was higher overall in Rol plants, particularly under control N, where it was approximately 20%–30% higher than in WT (Figure 3E). Starch concentration increased under N deficiency (by approximately 70%–80% at BOP) and was generally higher in WT than in Rol plants (Figure 3F).

Malate concentration was generally higher in Rol than in WT and increased under N deficiency (Figure 3G). Importantly, the EOP‐to‐BOP response depended on genotype and N supply, with malate concentration nearly doubling in WT under N deficiency, consistent with the significant T × *N* × G interaction. The effect of N supply on total flavonoid content likewise depended on sampling time, with concentrations approximately 30% higher under N deficiency at BOP but showing smaller differences at EOP (Figure 3H).

### 3.3 Rol Plants Maintain Higher Daytime Transpiration Under Nitrogen Deficiency

Leaf‐area‐normalized whole‐plant transpiration showed a clear diel pattern, with high rates during the light period and a strong decline during darkness (Figure 4). In WT plants, N deficiency reduced transpiration during most daytime measurements (Figure 4A). In Rol plants, daytime transpiration remained relatively high under both N regimes, and the difference between control and N‐deficient plants was smaller (Figure 4B). Overall, the descriptive diel profiles indicated a stronger reduction in daytime transpiration in N‐deficient WT than in Rol plants.

**FIGURE 4:**
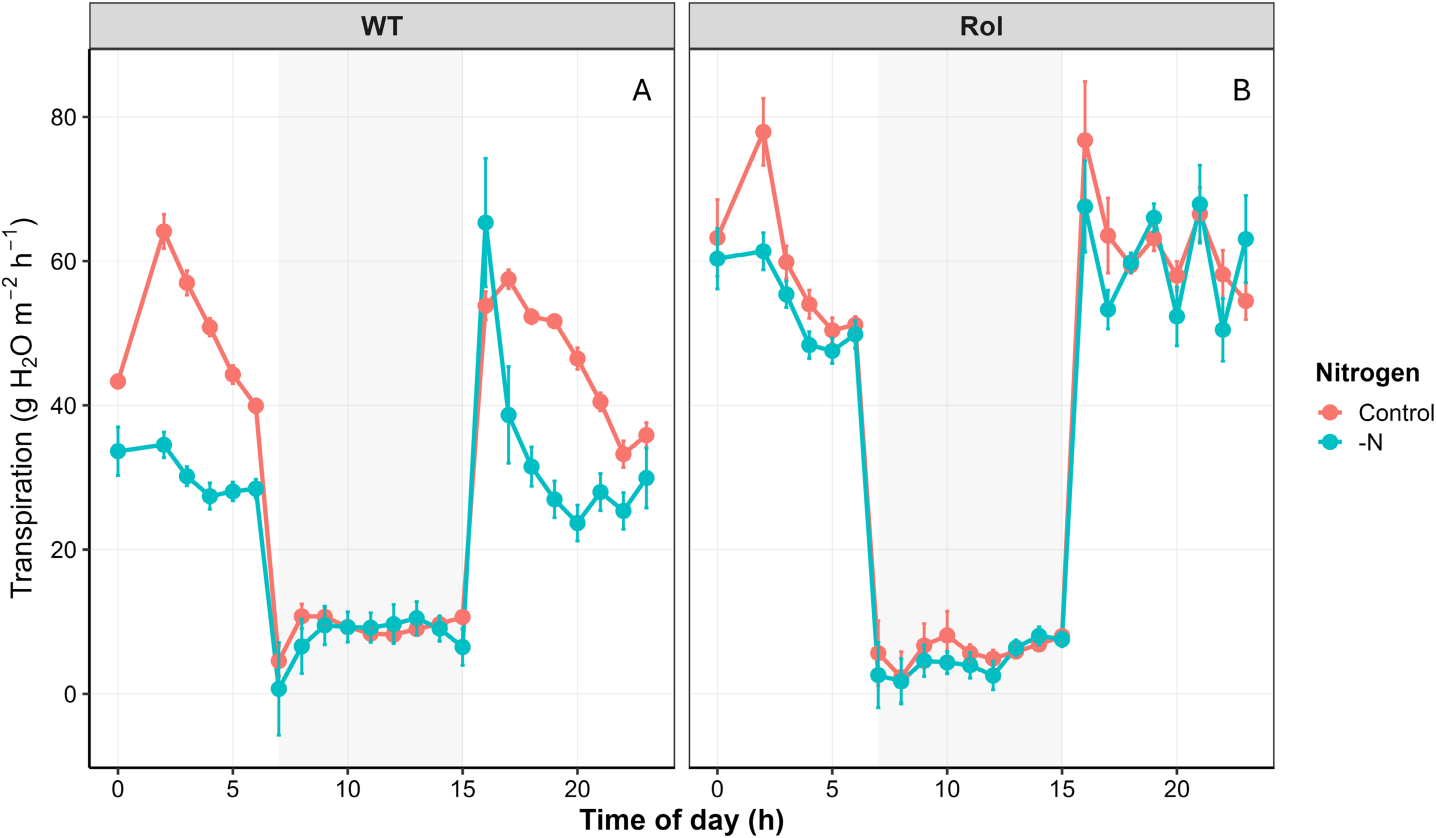
Effects of nitrogen supply and rol transformation on diel transpiration in the pot experiment. Transpiration was measured using the DroughtSpotter system, calculated from hourly pot weight loss and normalized to total leaf area. Panels show mean hourly transpiration rates across the diel cycle in WT (A) and Rol (B) plants grown under control and nitrogen‐deficient (−N) conditions. Values represent means ± SE from *n* = 6 biological replicates per treatment, calculated from the last 5 days before harvest. Shaded areas indicate the dark period.

### 3.4 Rol Plants Sustain Photosynthetic Activity Under Nitrogen Deficiency

Leaf gas exchange and photosynthetic performance differed with N supply and genotype (Figure 5). Net photosynthesis (P_n_) was strongly reduced by N deficiency and was lower overall in WT than in Rol plants. Although the *N* × G interaction was not significant, under N deficiency, P_n_ was nearly completely suppressed in WT plants, whereas Rol plants retained approximately 54% of their control photosynthetic rate (Figure 5A).

**FIGURE 5:**
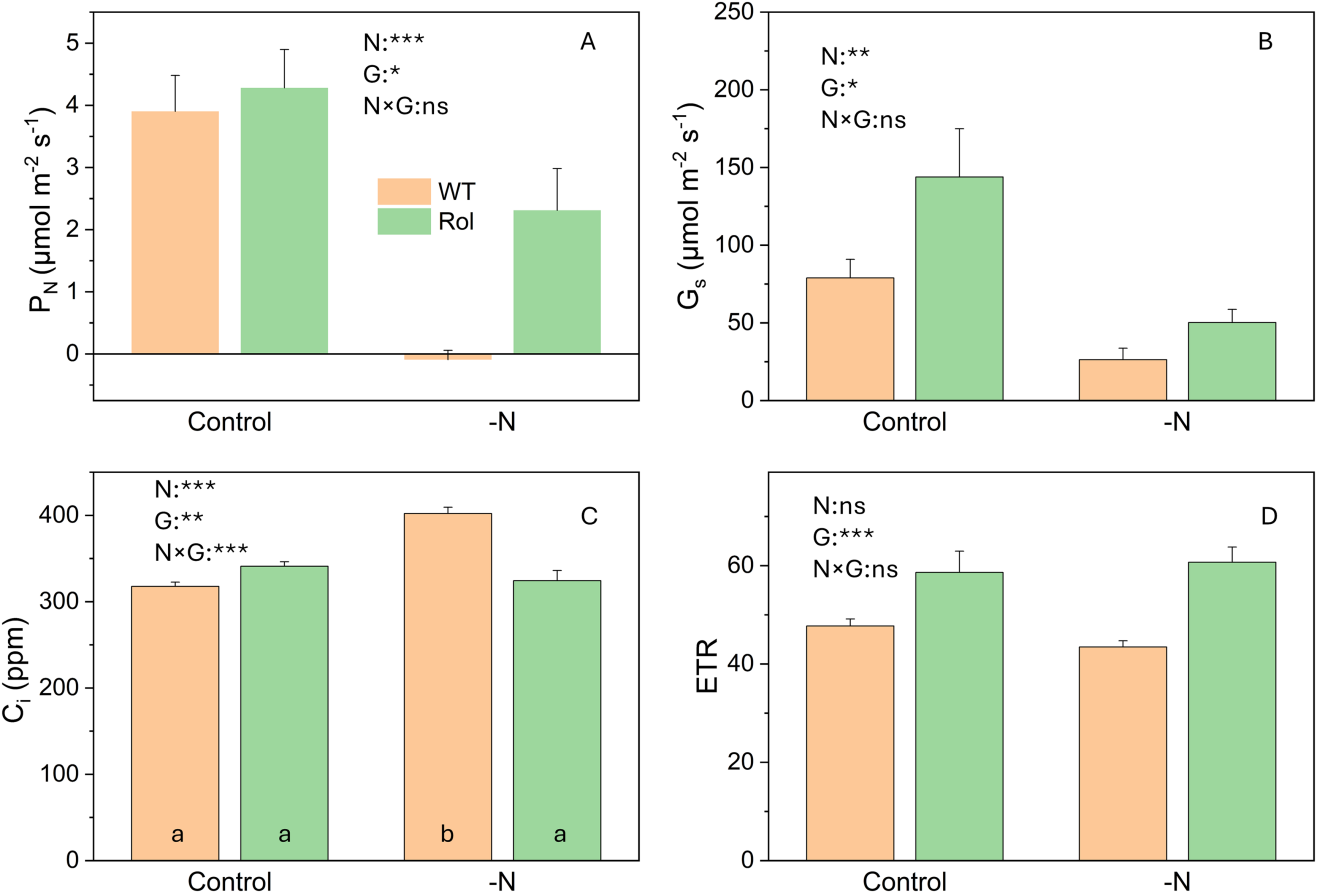
Effects of nitrogen supply and rol transformation on leaf gas exchange and photosynthetic performance in the pot experiment. Shown are net photosynthetic rate (P_n_) (A), stomatal conductance (g_s_) (B), intercellular CO_2_ concentration (Cᵢ) (C) and electron transport rate (ETR) (D) in *Kalanchoë*. WT and Rol plants were grown under control and nitrogen‐deficient (−N) conditions. Bars represent means ± SE (*n* = 6 biological replicates per treatment, except for −*N* Rol, where *n* = 5). Different lowercase letters indicate significant differences among treatment means at *p* < 0.05 by Tukey HSD only when the *N* × G interaction was significant; otherwise, inference is based on the ANOVA main effects.

Stomatal conductance was approximately 1.8‐fold higher overall in Rol than in WT plants (Figure 5B). In WT plants, N deficiency increased intercellular CO_2_ concentration by approximately 27%, reaching the highest values in this treatment (Figure 5C). Electron transport rate was approximately 30% higher overall in Rol than in WT plants, whereas N supply had no significant overall effect (Figure 5D).

### 3.5 Hydroponic Cultivation Confirms Major Architectural Traits of the Rol Phenotype and Reveals Enhanced Root Allocation

Hydroponic cultivation allowed tighter control of N availability and direct quantification of root biomass allocation (Figure 6). In contrast to the pot experiment, leaf area did not differ significantly between genotypes and was reduced by approximately 45% under N deficiency (Figure 6A). Total plant biomass showed a similar pattern, decreasing by approximately 23%–32% under N deficiency but not differing significantly between WT and Rol (Figure 6B). Thus, the lower leaf area and total biomass of Rol plants observed in pots were not observed under hydroponic conditions.

**FIGURE 6:**
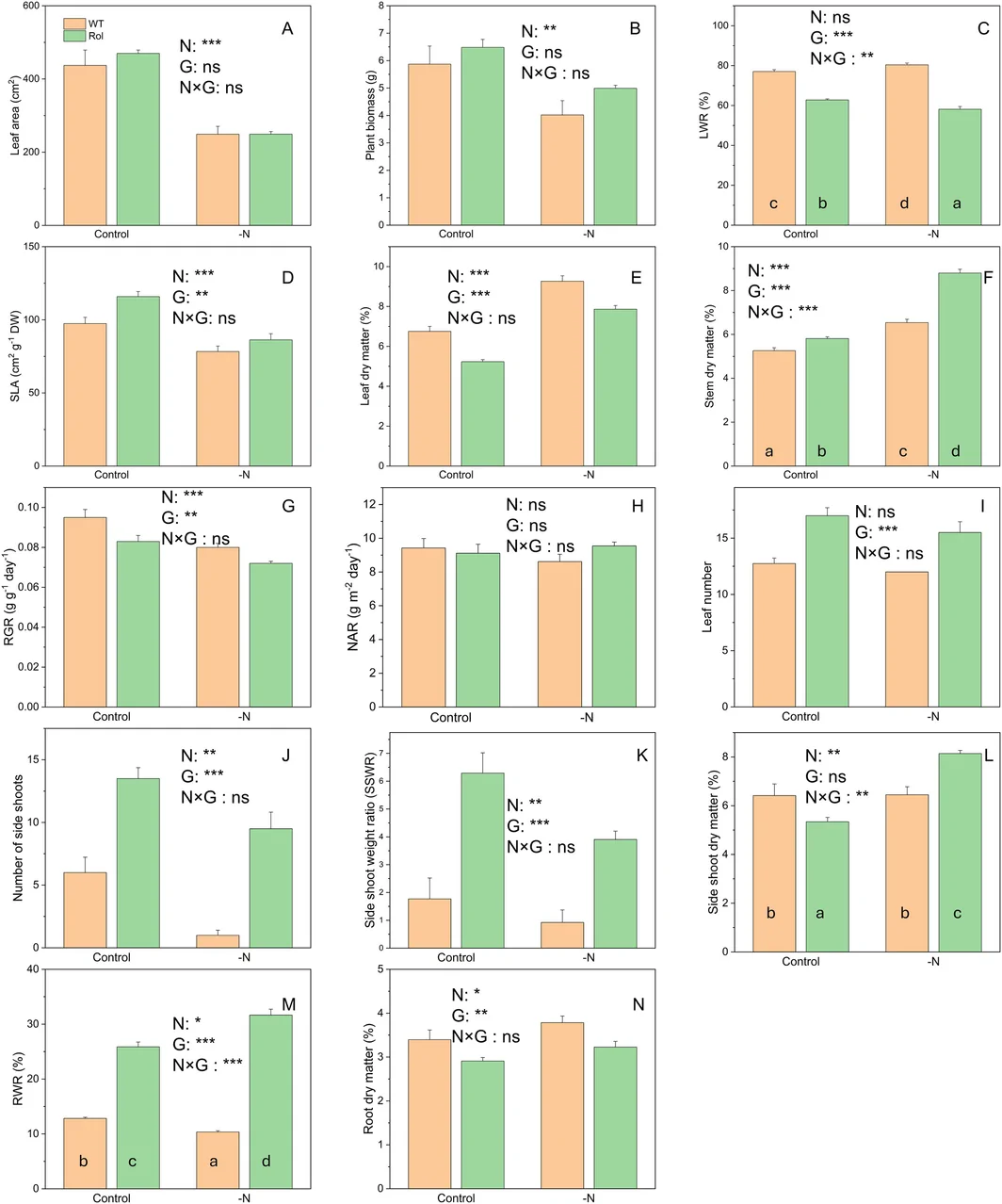
Effects of genotype and nitrogen supply on growth, biomass allocation and morphological traits in hydroponically grown *Kalanchoë* plants. WT and Rol plants were cultivated hydroponically under control nutrient supply or nitrogen deficiency (−N). Shown are leaf area (A), total plant biomass (B), leaf weight ratio (LWR) (C), specific leaf area (SLA) (D), leaf dry matter content (E), stem dry matter content (F), relative growth rate (RGR) (G), net assimilation rate (NAR) (H), leaf number (I), number of side shoots (J), side‐shoot weight ratio (SSWR) (K), side‐shoot dry matter content (L), root weight ratio (RWR) (M) and root dry matter content (N). Bars represent means ± SE (*n* = 4 biological replicates per treatment). Different lowercase letters indicate significant differences among treatment means at *p* < 0.05. Results of the two‐way ANOVA are shown in each panel: Nitrogen supply (N), genotype (G) and their interaction (*N* × G); ns, not significant; *p < 0.05, **p < 0.01, ***p < 0.001.

Several biomass‐allocation and leaf‐structural traits nevertheless confirmed the patterns observed in the pot experiment. Leaf weight ratio was approximately 18%–29% lower in Rol than in WT plants, with its response to N differing between genotypes (Figure 6C). Specific leaf area was 12%–20% higher in Rol, whereas leaf dry matter content was 15%–22% lower in Rol and increased by approximately 37%–50% under N deficiency (Figure 6D,E). Stem dry matter content also increased under N deficiency, by approximately 25% in WT and 52% in Rol (Figure 6F).

Relative growth rate (RGR) was approximately 8%–13% lower in Rol plants (Figure 6G), although this should be interpreted in the context of their larger initial biomass. Net assimilation rate did not differ significantly between genotypes or N treatments (Figure 6H). Rol plants produced approximately 30% more leaves than WT (Figure 6I) and also produced more side shoots (Figure 6J). Side‐shoot weight ratio was approximately fourfold higher in Rol (Figure 6K). N deficiency reduced side‐shoot number overall, while side‐shoot dry matter content increased by approximately 50% under low N in Rol plants but remained essentially unchanged in WT (Figure 6L).

The strongest genotype effect was observed for root biomass allocation. Root weight ratio was approximately twofold higher in Rol than in WT under control N and threefold higher under N deficiency; within Rol plants, it increased by approximately 25% under N deficiency (Figure 6M). Root dry matter content, by contrast, was approximately 15% lower in Rol than in WT and increased slightly under N deficiency (Figure 6N). Thus, hydroponic cultivation confirmed several characteristic features of the Rol phenotype, including higher SLA, lower leaf dry matter content, greater branching and, notably, substantially greater root allocation.

### 3.6 Hydroponic Analysis Largely Confirms Genotype‐Dependent Differences in Tissue Nitrogen Status, Pigments and Metabolites

In hydroponically grown plants, N deficiency strongly affected tissue N status, pigment composition and carbon‐related metabolites, while several genotype differences were consistent with those observed in the pot experiment (Figure 7).

**FIGURE 7:**
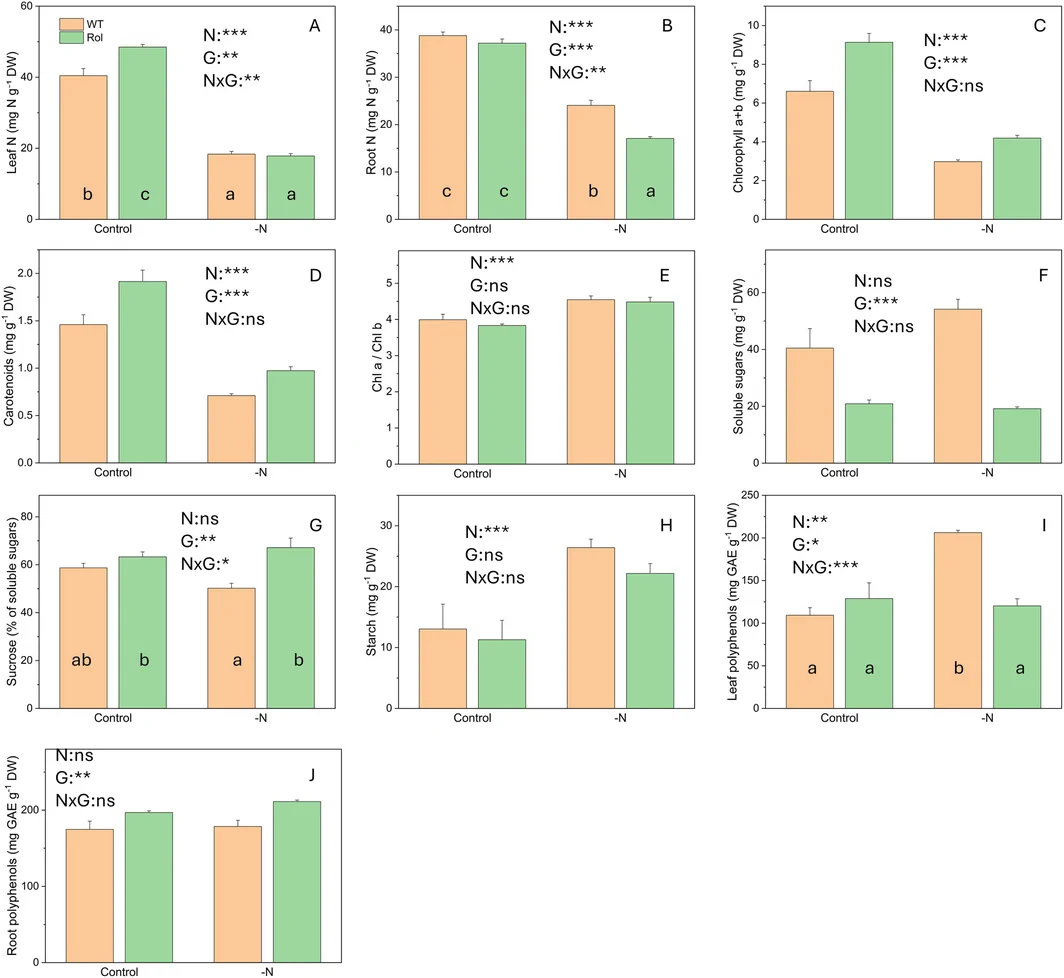
Effects of genotype and nitrogen supply on tissue nitrogen status, pigments and metabolites in hydroponically grown *Kalanchoë* plants. WT and Rol plants were cultivated hydroponically under control nutrient supply or nitrogen deficiency (−N). Shown are leaf N concentration (A), root N concentration (B), total chlorophyll concentration (chlorophyll a + b) (C), carotenoid concentration (D), chlorophyll a/b ratio (E), leaf soluble sugars (F), sucrose as a proportion of total soluble sugars (G), starch concentration (H), leaf polyphenol concentration (I) and root polyphenol concentration (J). Bars represent means ± SE (*n* = 4 biological replicates per treatment). Different lowercase letters indicate significant differences among treatment means at *p* < 0.05 by Tukey HSD only when the *N* × G interaction was significant; otherwise, inference is based on the ANOVA main effects. Results of the two‐way ANOVA are shown in each panel: Nitrogen supply (N), genotype (G) and their interaction (*N* × G); ns, not significant; *p < 0.05, **p < 0.01, ***p < 0.001.

Leaf N concentration decreased by approximately 54% in WT and 64% in Rol under N deficiency. Under control N, leaf N was approximately 20% higher in Rol than in WT, whereas this genotype difference disappeared under low N (Figure 7A). Root N concentration also decreased under N deficiency by approximately 38% in WT and 54% in Rol. Under control N, the genotypes did not differ, whereas under low N root N concentration was approximately 40% higher in WT than in Rol (Figure 7B).

Pigment responses closely resembled those observed in the pot experiment. Total chlorophyll and carotenoid concentrations decreased by approximately 55% and 50%, respectively, under N deficiency, but remained higher in Rol than in WT under both N regimes (Figure 7C,D). By contrast, the chlorophyll *a*/*b* ratio increased by approximately 14% under N deficiency but was not affected by genotype (Figure 7E).

Leaf soluble sugar concentration was approximately 55% lower overall in Rol than in WT, whereas N supply had no significant overall effect (Figure 7F). Under N deficiency, the proportion of sucrose in total soluble sugars was approximately 35% higher in Rol than in WT, whereas the genotypes did not differ under control N (Figure 7G). Starch concentration approximately doubled under N deficiency in both genotypes (Figure 7H).

Polyphenol accumulation differed between leaves and roots. In leaves, polyphenol concentration increased by approximately 87% in WT under low N but remained essentially unchanged in Rol, resulting in a significant *N* × G interaction (Figure 7I). In roots, polyphenol concentration was not affected by N supply but was approximately 14% higher overall in Rol than in WT (Figure 7J).

### 3.7 Hormonal Profiling Reveals Constitutive Genotype Effects and Organ‐Specific Nitrogen Responses

Hormone profiles revealed strong genotype‐dependent differences, while responses to N deficiency varied among hormones and organs (Figure 8). ABA concentration was approximately 70% lower in leaves and 50% lower in roots of Rol than WT plants and was not significantly affected by N supply (Figure 8A,B). PA, the major ABA catabolite, showed the same genotype pattern, being approximately 30% lower in Rol leaves and 35% lower in Rol roots and was likewise unaffected by N supply (Figure 8C,D).

**FIGURE 8:**
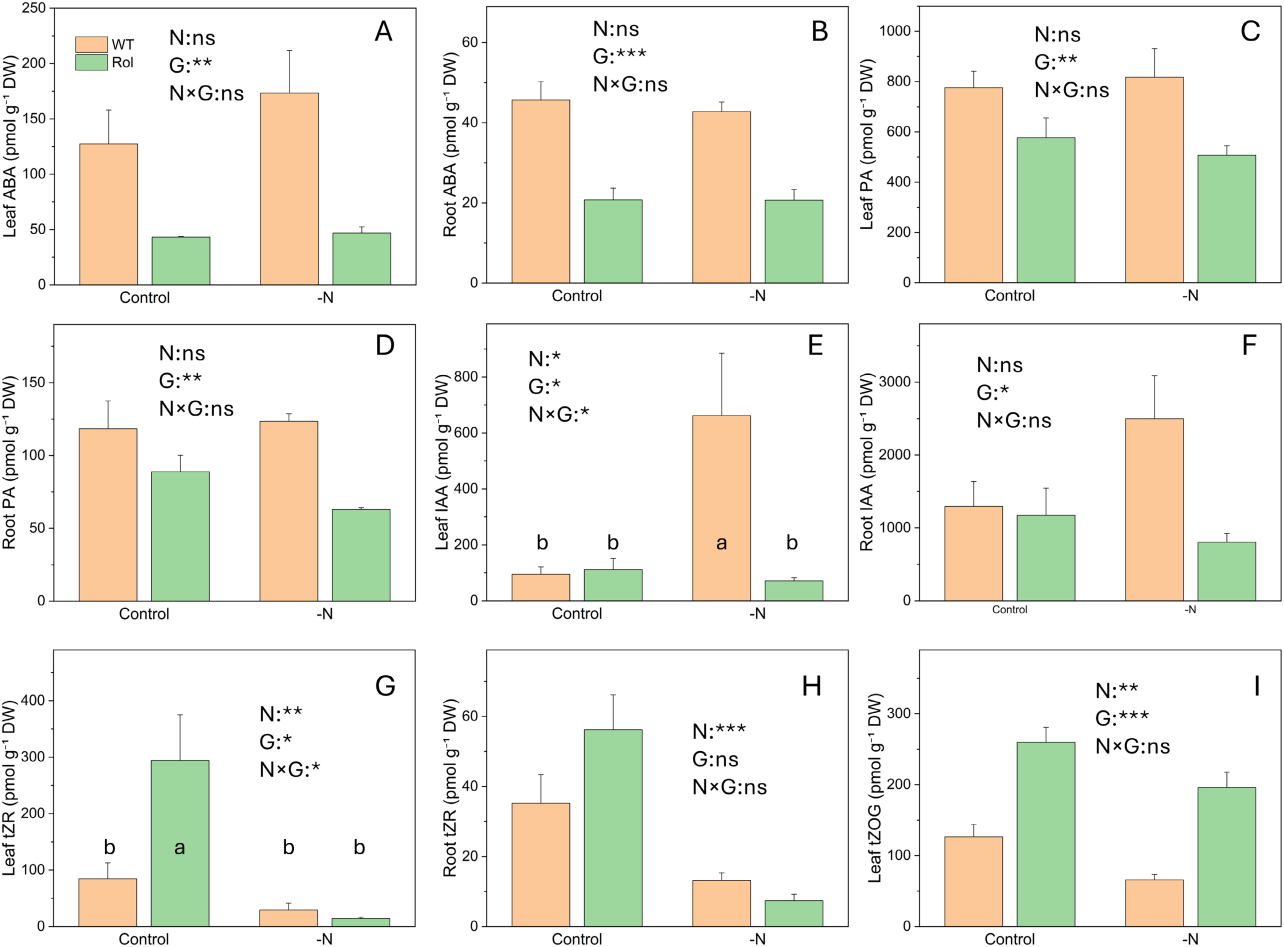
Effects of genotype and nitrogen supply on ABA‐, auxin‐ and cytokinin‐related pools in leaves and roots of hydroponically grown *Kalanchoë* plants. Shown are concentrations of abscisic acid (ABA) in leaves (A) and roots (B), phaseic acid (PA) in leaves (C) and roots (D), indole‐3‐acetic acid (IAA) in leaves (E) and roots (F), trans‐zeatin riboside (tZR) in leaves (G) and roots (H) and trans‐zeatin O‐glucoside (tZOG) in leaves (I) of wild‐type (WT) and rol‐transformed (Rol) plants grown hydroponically under control nutrient supply or nitrogen deficiency (−N). Bars represent means ± SE (*n* = 4 biological replicates per treatment). Different lowercase letters indicate significant differences among treatment means at *p* < 0.05 by Tukey HSD only when the *N* × G interaction was significant; otherwise, inference is based on the ANOVA main effects. Results of the two‐way ANOVA are shown in each panel: Nitrogen supply (N), genotype (G) and their interaction (*N* × G); ns, not significant; *p* < 0.05, *p* < 0.01, *p* < 0.001. Hormone concentrations are expressed on a dry weight basis (pmol g^−1^ DW).

IAA responses differed between organs. In leaves, IAA increased approximately sevenfold under N deficiency in WT plants but showed no corresponding increase in Rol, resulting in a significant *N* × G interaction (Figure 8E). In roots, IAA was lower overall in Rol than in WT plants, with the largest numerical difference observed under N deficiency (Figure 8F).

Cytokinin‐related traits also differed between genotypes. Under control conditions, leaf tZR was approximately 3.5‐fold higher in Rol than in WT, whereas this genotype difference disappeared under N deficiency, consistent with the significant *N* × G interaction (Figure 8G). In roots, tZR decreased by approximately 75% under N deficiency but was not significantly affected by genotype (Figure 8H). Leaf tZOG was approximately 2.3‐fold higher overall in Rol than in WT and decreased by approximately 30% under N deficiency, without a significant *N* × G interaction (Figure 8I).

Overall, Rol plants were characterized by lower ABA, PA and root IAA concentrations together with higher leaf cytokinin‐related pools than WT.

## 4 Discussion

Nitrogen deficiency strongly restricted growth in both WT and Rol plants, confirming the importance of N availability for biomass production in *Kalanchoë*. Across both experiments, Rol plants consistently displayed a distinct phenotype characterized by stronger branching, higher specific leaf area, lower leaf dry matter content, greater root allocation and altered carbon partitioning. In the hydroponic experiment, Rol plants also showed a markedly different hormonal profile. Under N deficiency, Rol plants also maintained positive net photosynthesis, higher pigment concentrations and higher electron transport, whereas WT plants showed an almost complete suppression of CO_2_ assimilation. Together, these findings suggest that rol transformation did not simply modify plant morphology, but was associated with broader changes in the developmental and physiological context through which *Kalanchoë* responded to nitrogen limitation.

### 4.1 Rol Transformation Is Associated With Altered Plant Architecture and Stronger Root Investment

A central and highly consistent outcome of this study was that rol transformation was associated with altered plant architecture under both sufficient and limiting N supply (Figures 1, 2 and 6). In both cultivation systems, Rol plants produced more leaves and side shoots than WT plants and showed higher specific leaf area (Figures 2D and 6D) together with lower leaf dry matter content (Figures 2E and 6E). These traits define a compact but highly branched phenotype and indicate altered biomass distribution among organs.

The belowground response was equally pronounced. In the pot experiment, Rol plants showed visibly stronger root development, while in hydroponics, this was confirmed quantitatively by the much higher root weight ratio of Rol plants under both N regimes (Figure 6M). Enhanced root development therefore appears to be a stable feature of the Rol phenotype. N deficiency commonly promotes a relative shift in biomass allocation towards roots, although the magnitude and even direction of this response vary among species, genotypes and experimental conditions. Importantly, an increase in root weight ratio under low N does not necessarily imply greater absolute root biomass, but may instead reflect stronger suppression of shoot growth together with altered carbon allocation. N limitation can also modify root morphology and architecture without increasing total root biomass (Lopez et al. 2023).

In this context, the contrasting response of the two genotypes is notable. Rol plants showed strong root investment already under sufficient N and a further increase in root allocation under low N, whereas WT plants showed no corresponding increase. Thus, rol transformation was associated not only with constitutively greater root investment but also with greater plasticity of biomass allocation in response to N limitation. Together with the lower leaf weight ratio (Figure 6C) and enhanced lateral shoot formation (Figure 6J), this indicates that rol transformation altered whole‐plant allocation rather than individual organs in isolation. Such plastic shifts in root investment may be relevant to improve nutrient foraging under restricted nutrient availability (Bhattacharya et al. 2026).

The functional consequences of this altered allocation differed between cultivation systems. In the pot experiment, Rol plants had lower leaf area and total biomass than WT plants (Figure 2A,B), particularly under non‐limiting N supply, whereas in hydroponics these genotype differences disappeared (Figure 6A,B). Because enhanced root development was evident in both systems, this contrast is unlikely to reflect a system‐specific root phenotype. One possible explanation is that the root‐oriented growth strategy of Rol plants was more strongly constrained in pots, where root expansion was physically restricted, whereas hydroponics allowed the characteristic Rol phenotype to develop without the same reduction in growth. Thus, Rol plants may not be intrinsically smaller than WT plants, but may depend more strongly on sufficient rooting space to express their growth potential. This possibility also suggests that conventional pot systems could underestimate the performance of genotypes with intrinsically high root investment.

### 4.2 Rol Plants Appear to Maintain Photosynthetic Function Under Nitrogen Deficiency, Potentially Through Altered Source‐Sink Balance and CAM Regulation

A second major outcome was the markedly different photosynthetic status of the two genotypes under N deficiency. Although the *N* × genotype interaction for net photosynthesis was not significant, WT plants showed an almost complete loss of net CO_2_ assimilation under low N, whereas Rol plants maintained positive photosynthesis (Figure 5A). Rol plants also maintained higher total chlorophyll and carotenoid concentrations (Figures 3A,C and 7C,D) and higher electron transport rate (Figure 5D), indicating better preservation of light harvesting and photochemical activity. However, these responses should not be interpreted solely in terms of stability of the photosynthetic apparatus. Plant performance depends on source activity together with assimilate transport and sink utilization and source and sink functions frequently co‐limit growth (Sonnewald and Fernie 2018; Fernie et al. 2020).

The gas‐exchange data further suggest that the severe reduction in photosynthesis in WT under low N was not primarily caused by stomatal limitation. Intercellular CO_2_ concentration was highest in WT plants under low N (Figure 5C), indicating that CO_2_ diffusion into the leaf was unlikely to be the dominant constraint. Instead, the near‐complete loss of net assimilation despite high Ci is consistent with strong non‐stomatal limitation, potentially involving biochemical CO_2_ fixation and/or downstream photosynthetic metabolism. In this context, the higher pigment concentrations and electron transport rate observed in Rol plants are consistent with better maintenance of photosynthetic capacity. However, the metabolite data suggest that this may also have depended on stronger sink activity and more effective carbon utilization, rather than on preservation of the photosynthetic apparatus alone.

Differences in transpiration were consistent with this physiological pattern. The diel profiles showed that N deficiency reduced daytime transpiration more clearly in WT than in Rol plants (Figure 4A,B), paralleling the strong decline in net photosynthesis in WT under low N (Figure 5A). Rol plants maintained comparatively high daytime transpiration under N deficiency (Figure 4B), in agreement with their generally higher stomatal conductance (Figure 5B). Because *Kalanchoë blossfeldiana* and *Kalanchoë laciniata* are CAM species, these diel gas‐exchange patterns may reflect not only quantitative differences in stomatal opening but also differences in the temporal organization of carbon fixation. In CAM plants, malate produced during the dark period is subsequently decarboxylated during the day to supply CO_2_ near Rubisco, requiring coordinated regulation of enzymes, transport processes and stomatal behaviour (Yang et al. 2015; Borland et al. 2016; Yuan et al. 2020).

CAM expression varies considerably among species and environments, ranging from facultative to constitutive CAM and differing in the relative contribution of individual CAM phases (Winter and Holtum 2014; Winter 2019; Yuan et al. 2020). Nitrogen availability can also affect CAM magnitude, including nocturnal CO_2_ uptake, PEPC activity and organic‐acid accumulation in Kalanchoë (Pereira and Cushman 2019).

Perturbation of CAM‐related components can likewise alter the temporal pattern of CO_2_ fixation, including loss of dark CO_2_ fixation or shifts in late‐day and nocturnal carbon uptake (Dever et al. 2015; Boxall et al. 2017; Boxall et al. 2020; Liu et al. 2019). The present genotype differences may therefore reflect differential maintenance of photosynthetic capacity under N deficiency and may also be associated with differences in diel carbon turnover and CAM phase coordination. Direct measurements of nocturnal CO_2_ uptake would be required to distinguish these possibilities.

Malate adds an important dimension to this interpretation. Malate concentration was generally higher in Rol plants and increased under N deficiency (Figure 3G), while the EOP–BOP response depended on genotype and N supply and was particularly pronounced in WT under low N. In a CAM species, such diel differences are directly relevant to the temporal coordination of carbon fixation and decarboxylation. Because malate links nocturnal carbon capture with daytime CO_2_ release, these patterns raise the possibility that rol transformation modified diel carbon turnover under N deficiency. This interpretation is consistent with previous work showing that N availability and N source can influence nocturnal malate accumulation and CAM expression in *Kalanchoë* (Pereira and Cushman 2019). However, these possibilities should be regarded as working hypotheses rather than direct conclusions from the present dataset.

The carbon‐metabolite data also support a broader source–sink interpretation. In both experiments, Rol plants showed lower total soluble sugar concentrations than WT plants (Figures 3D and 7F), but a higher sucrose/TSS ratio (Figures 3E and 7G). This pattern may indicate that carbon in Rol plants was maintained more in transport form than in the broader soluble carbohydrate pool, consistent with stronger branching and greater root development, thereby sustaining sink demand. Starch accumulated under low N in both experiments (Figures 3F and 7H) and, in the pot experiment, was generally higher in WT than in Rol plants (Figure 3F). Because N limitation can restrict growth and carbon utilization more strongly than carbon capture, the greater accumulation of soluble sugars in WT, together with its higher starch concentrations in the pot experiment, may reflect weaker sink utilization rather than a more favourable carbon status. Conversely, the lower carbohydrate accumulation, higher sucrose/TSS ratio and stronger root allocation of Rol plants are consistent with continued assimilate export towards active sinks (Sonnewald and Fernie 2018; Fernie et al. 2020; Rosado‐Souza et al. 2023).

Overall, the carbon and gas‐exchange data suggest that Rol plants maintained a different balance among photosynthetic activity, sink demand and carbon utilization under N deficiency, potentially involving stronger source‐sink coupling and altered CAM‐associated diel carbon metabolism. Direct measurements of CAM phase behaviour and assimilate flux will be required to establish the underlying mechanisms.

### 4.3 Hormonal Changes Support, but Do Not Fully Explain, the Rol Phenotype

Hormonal profiling revealed a marked shift in hormonal balance associated with rol transformation. Rol plants were characterized by lower ABA and PA concentrations, lower auxin status, particularly in roots, and higher cytokinin‐related pools in leaves (Figure 8). Because several of these differences were already evident under control conditions, rol transformation appears to have altered the regulatory background of the plant rather than simply modifying its response to low N. At the same time, genotype‐dependent responses of leaf IAA and tZR indicate that the hormonal response to N limitation was also modified.

The lower ABA and PA concentrations in Rol plants are noteworthy in view of their higher daytime transpiration and tendency towards higher stomatal conductance. ABA is a central regulator of stomatal closure and water‐conserving stress responses, and reduced ABA status may therefore have contributed to the maintenance of daytime gas exchange in Rol plants (Wilkinson and Davies 2002; Cutler et al. 2010). However, this interpretation remains associative because stomatal sensitivity to ABA was not directly tested.

Auxin‐related responses also differed between genotypes. Root IAA concentration was lower in Rol than in WT plants, while leaf IAA increased under N deficiency in WT but showed no corresponding increase in Rol (Figure 8E,F). Given the central role of auxin in root–shoot patterning, apical dominance, branching and lateral organ development, altered auxin status is consistent with several characteristics of the Rol phenotype (Teale et al. 2006; Domagalska and Leyser 2011). *Rol* genes are widely associated with altered auxin sensitivity or signalling, supporting modified auxin regulation as one component of the transformed phenotype (Bulgakov 2008). Nevertheless, the present data do not support direct one‐to‐one relationships between bulk auxin concentration and individual traits.

Cytokinin‐related traits also differed between genotypes. Leaf tZR was higher in Rol under sufficient N and declined under N deficiency, whereas tZOG remained higher in Rol than in WT across N regimes (Figure 8G,I). Cytokinins are closely linked to shoot activity, chlorophyll retention, leaf function and systemic signalling of N availability (Sakakibara 2006; Kiba et al. 2011; Poitout et al. 2018). The enhanced cytokinin status of Rol plants may therefore be associated with their higher chlorophyll and carotenoid concentrations and maintenance of leaf function. Taken together, however, the hormone data support a distinct regulatory background rather than a simple mechanism based on changes in any single hormone pool.

### 4.4 A Whole‐Plant Working Model for the Rol Response to Low Nitrogen

Overall, the results suggest that rol transformation modified the response to N deficiency at the whole‐plant level. The Rol phenotype appears to represent a constitutively altered developmental and physiological state on which N limitation acted, rather than an amplification of a standard low‐N syndrome. This view is broadly consistent with models in which plant responses to N limitation arise from integrated root–shoot signalling involving auxin, cytokinin and ABA (Ruffel et al. 2011; Jia et al. 2022).

A central feature of the Rol phenotype was stronger root investment together with enhanced shoot branching, yet these traits were not directly reflected by corresponding changes in bulk IAA, cytokinins, or ABA concentrations. In model plants, N‐dependent changes in root architecture involve auxin‐, cytokinin‐ and ABA‐related signalling, including NRT1.1‐mediated auxin transport, TAR2‐dependent auxin biosynthesis and cytokinin‐mediated root‐to‐shoot signalling (Krouk et al. 2010; Ma et al. 2014; Takei et al. 2002; Takei et al. 2004; Poitout et al. 2018; Signora et al. 2001; Ondzighi‐Assoume et al. 2016). The present results suggest that rol transformation may instead affect hormone sensitivity, transport, compartmentation, or downstream integration rather than simply changing bulk hormone concentrations.

For comparison, N deficiency increased the root weight ratio in tomato (Paponov et al. 2021), consistent with the common shift towards greater root allocation under low N. In the present study, however, the root‐oriented phenotype of Rol plants was already evident under sufficient N and increased further under low N, whereas WT plants showed no corresponding increase. Rol transformation therefore does not appear simply to amplify a standard N‐deficiency response but may redirect it through a distinct developmental and regulatory framework.

Within this framework, stronger root development could increase sink activity and resource capture, while the maintained leaf function could sustain carbon assimilation and export.

The contrast between pot and hydroponic cultivation further suggests that the functional value of this phenotype depends on the growth environment. Although enhanced root development was evident in both systems, lower biomass of Rol plants was observed in pots but not in hydroponics (Figures 2B and 6B). When root growth is physically restricted, strong root investment may therefore constrain aboveground growth, whereas unrestricted root development may allow the same phenotype to be expressed without an overall biomass penalty.

The compact Rol phenotype also represents a developmental route distinct from classical Green Revolution dwarfing, which largely involves GA biosynthesis or DELLA‐related signalling and can influence nitrogen‐use efficiency (Li et al. 2018). In contrast, rol transformation was associated here with strong root investment and altered carbon economy.

## 5 Limitations and Perspectives

Although the present study identifies clear genotype‐dependent differences in morphology, physiology, metabolism and hormonal status under N deficiency, several limitations should be considered. First, the data reveal associations between hormonal balance and plant performance but do not establish a direct causal relationship. Although the lower ABA‐ and auxin‐related pools and higher cytokinin status of Rol plants are consistent with their altered architecture and physiological phenotype, the contribution of these hormonal changes remains unresolved. Future work should therefore determine more directly how *rol* genes influence ABA‐, auxin‐ and cytokinin‐related regulation under contrasting N conditions.

A second limitation concerns diel carbon metabolism. Because *Kalanchoë* is a CAM species, the contrasting patterns of daytime transpiration and malate accumulation suggest possible genotype‐dependent differences in CAM‐related regulation under N deficiency. However, nocturnal CO_2_ uptake, higher‐resolution diel malate turnover and phase‐specific CAM behaviour were not measured. Such analyses will be necessary to determine whether WT and Rol plants differ in CAM expression under low N and to what extent rol transformation modifies the balance between daytime assimilation and CAM‐related carbon fixation. Given the markedly greater root allocation of Rol plants, future studies should also determine whether rol transformation alters the uptake and tissue distribution of sulphur and other mineral nutrients.

Finally, the contrasting results obtained in pot and hydroponic culture highlight the need to test directly how rooting volume and physical growth environment influence expression of the Rol phenotype. Rol plants may be more sensitive to root confinement than WT plants and may require a larger effective rooting volume to express their growth potential. It will therefore be important to examine whether increased root allocation translates into improved N uptake kinetics, greater N acquisition capacity, or altered nitrogen use efficiency when root growth is not restricted. Such experiments would help clarify whether the stronger root system primarily affects nutrient acquisition, biomass partitioning, or both and would test its possible value in applications where extensive root development is advantageous. Overall, the Rol phenotype provides a useful framework for investigating how developmental and hormonal changes interact with nitrogen supply and cultivation environment in CAM plants.

## Author Contributions

I.A.P. conceived and designed the study, analysed the data and wrote the manuscript. A.E. carried out the pot and hydroponic experiments, performed growth and pigment analyses and contributed to data analysis. G.S. performed the chemical analyses and analysed the corresponding data. Y.V.E.K. performed the photosynthetic measurements and contributed to the analysis, interpretation and description of these results. N.P.V. performed the sugar analyses. O.V. and P.T. performed the hormone analyses. M.P., J.G. and K.L.N. provided the plant material and contributed to the experimental design. All authors reviewed and approved the final manuscript.

## Funding

This work was supported by Innovation Fund Denmark (Innovationsfonden; case no. 3109‐00058B) and co‐funded by the European Union through the Horizon Europe programme under the Eurostars‐3 partnership (project E2724 GREENPLANT).

## Data Availability

The data that support the findings of this study are available within the article.
